# Indications for and Method of Operating upon the Middle Turbinated Bone

**Published:** 1900-04

**Authors:** John P. Davidson

**Affiliations:** Professor of Diseases of the Eye, Ear and Throat, Medical College of Virginia, Richmond, Va.


### INDICATIONS FOR AND METHOD OF OPERATING UPON THE MIDDLE TURBINATED BONE.

By JOHN P. DAVIDSON,

Professor of Diseases of the Eye, Ear and Throat, Medical College of Virginia, Richmond, Va.

The indications which cause a demand for operative procedure upon the middle turbinated bone may be briefly summarized as follows:

1. For a large and well-defined class of cases suffering from nasal obstruction.

2. To remove an etiological factor preparatory for the further treatment of naso-pharyngitis and chronic laryngitis.

3. For the relief obtained in certain cases of chronic catarrhal otitis media.

4. For the beneficial effects procured in cases suffering from reflex nervous symptoms, either accompanied or unaccompanied by asthma or hay fever.

It will be observed that the foregoing conditions usually accompany hypertrophic rhinitis—thickening of the mucous membrane lining the nasal chambers. There are, however, important exceptions to this, in that the atrophic condition of the mucous membrane is sometimes accompanied by enlargement of the bony structure of the middle turbinate.

In considering surgical intervention for the first class of cases, viz., those suffering from nasal obstruction, it is not intended to give equal prominence to turbinate hypertrophy with that of abnormalities of the nasal septum. Surely no factor figures so conspicuously in the causation of intra-nasal thickening as deformities and deflections of the nasal septum, either cartilaginous or bony—more particularly the cartilaginous variety; and the existence of such lesions is undoubtedly the first problem presented to us for consideration.

Deflections should be straightened—deformities removed so far as it is possible, but it must be admitted that irregularities and zigzag conditions of the septum are often presented to us which no known method of treatment can relieve. The ill effects of such septa are, nevertheless, continuously being wrought, resulting in hypertrophic rhinitis of varying degrees.

It is found that this hypertrophy involves the turbinated bones, and that the greatest amount of enlargement occurs at corresponding concavities on the septum. It is further observed that the hypertrophy of the middle and inferior turbinates are not of a similar character. In the middle turbinate the bony framework is concerned in the hypertrophy, which is shown by an increased resistance to pressure by the probe as compared with the lower turbinate. It presents a less congested appearance, a more regular surface, and usually secretes less.

When we are forced to obtain an increased breathing space by some method to lessen the bulk of the turbinates, it seems to me that a number of reasons may be given why it is more advantageous to attack the middle in preference to the lower turbinate.

In the first place, the middle body possesses comparatively little function in comparison to that of the inferior. In the second place, the location of the middle body above the inferior is such that congestion in this structure will settle to the inferior by gravitation when the patient is standing, just as congestion settles to one side of the nose when the patient lies on the side. This can be readily observed in those patients who have had the inferior turbinates repeatedly cauterized with a recurrence of the congestion, the same cases presenting an entirely different appearance in the lower turbinate after having the hypertrophy of the middle bone removed. It is amazing to see how quickly the lower turbinate will lessen in its size with no treatment other than cleanliness after removal of a portion of the middle turbinate. In the third place, when the anterior portion of the bone is enlarged it prevents a proper drainage from the meatus and sinuses above, and the secretions from these cavities are drained, by the projection posteriorly of the middle turbinate, into the vault of the pharynx. This would be an obstructive lesion demanding relief which would not be complained of by the patient as an interference with breathing, though the drainage posteriorly of the secretions may be offered as a complaint.

It may be necessary to remove a portion of the middle turbinate to procure better drainage in cases of disease of any of the accessory cavities. The close apposition of this bone to the openings of the several sinuses renders it susceptible of being secondarily involved, and, as a matter of fact, we frequently find it softened, enlarged, and in a condition of so-called polypoid degeneration from its being secondarily involved by diseased conditions of the accessory cavities. By its removal in those cases drainage is facilitated and a better access is gained to the diseased region and more efficient treatment instituted.

The second class of cases in which diseases of the naso-pharynx and larynx require a partial removal of the middle turbinate are of equal importance with those accompanying obstructive lesions in the nose. It is a well-recognized clinical fact that naso-pharyngeal catarrh, together with chronic laryngitis, is frequently the result of diseased conditions or anomalous formations of the nasal chambers. Admitting this fact, it is not difficult to see why the enlarged middle turbinate figures so conspicuously as an etiological factor in the production of these secondary conditions, when we recall the anatomical location of the middle body and observe that its enlargement and projection posteriorly renders it a natural drainage for the secretions of the superior meatus and sinuses of the nose back into the vault of the pharynx. And, moreover, projection of the turbinate in a shelf-like manner from its attachment toward the septum, and slanting as it does from above downward, from before backward, prevents the natural secretion from passing over the turbinate and directs it into the vault of the pharynx. An ideal arrangement is therefore established for the drainage of the upper portion of the nasal chambers in the posterior direction. This method of drainage is made perfect when the increase in the size of the bony structure is sufficient to make pressure against the septum throughout its extent from before backward. The constant dripping of these more or less pathological secretions into the vault of the pharynx must add to whatever other causative factors which exist there to produce such an inflammation.

Just why chronic laryngitis ensues upon chronic naso-pharyngitis cannot be exactly explained. Perhaps the constant effort made to clear the throat, caused by the constant dropping from the vault of the pharynx, may produce an irritation and cough which, when kept up for a period of time, will produce an established chronic laryngitis. The dripping from the projecting end of the turbinate may fall in the region of the epiglottis and produce a mechanical irritation which extends into the larynx.

Rhinoscopic examination frequently reveals the enlarged and projecting turbinate covered by its own increased and perverted secretion, together with that collected from the cavities above. After the removal of these secretions by the employment of the post-nasal douche, the middle turbinate presents a pale, flabby appearance from its long-continued congestion.

The third, and perhaps the most important, class of cases demanding the removal of a portion of the middle turbinate, is the progressive impairment of hearing due to chronic catarrh of the middle ear. I am prepared to hear this statement criticized, but I

can append the history together with the functional testing of a number of cases operated upon during the last eighteen months, which it seems to me, would bear out this assertion. It is indeed striking to see how frequently enlargement of the middle turbinated bones occurs in conjunction with chronic catarrhal otitis media cases. When we consider the relation of the bone in question to the opening of the Eustachian tube, particularly when the size of the bone is increased at its posterior extremity, we observe its location to be just anterior and above the tubal aperture. Now, then, an inflamed or congested condition of the posterior extremity of the bone would have the same tendency to produce congestion by gravitation about the opening of the tube that it has in its anterior portion to cause hyperemia of the inferior turbinate. The existence of inflammation of the mucous membrane here will necessarily extend into the tube and along its caliber to the middle ear.

Even when actual inflammation of the lining membrane of the tube does not take place, the engorgement produced by the enlarged end of the turbinate interferes with the return circulation from the middle ear. The reason for the improvement of hearing is perhaps not the same in all cases. In some instances the removal of the mechanical obstruction caused by the bone is the source of relief. The improvement of a localized inflammation in this region accounts for the benefit in other cases. But what is most observed clinically is a general improvement obtained in the condition of the upper air-passages as a whole. In speaking of the improvement of hearing in these cases as a class, it may be stated that the forced whisper is heard at greater distance, as is also the watch, and that the lower tone limit is reduced from the point to which it had been raised. They have all been cases which possessed normal, or practically normal, hearing for the high tones, so that no change has occurred for the Galton whistle. It is furthermore fair to state that the other usual methods of treating these cases have not been suspended, though surely the effect cannot be attributed to this alone, since some of them have been cases which have been treated vigorously from time to time.

The fourth class of cases comprises those which are afflicted by neurotic symptoms, either accompanied or unaccompanied by asthma or hay fever.

I have to confess that I have not been impressed with the frequency of headache produced by the pressure of the enlarged middle turbinate against the septum which many observers report. I believe that many such cases can be traced to latent muscular and refractive errors. It is by no means intended to dispute the existence of such cases, but to convey the idea that they are not as frequent as is generally supposed. The fact that such headaches are relieved temporarily by the application of cocain does not prove them to be pressure headaches. Cocain will relieve headache from other causes temporarily, so that it cannot be relied upon as conclusive evidence in establishing the presence of pressure headaches. On the other hand, the reflex nervous symptoms giving rise to asthma and hay fever are by no means rare, and surely constitute a very important class of cases demanding surgical intervention in the nose, and especially upon the structures of the middle turbinated bones.

*Methods of Operating upon the Middle Turbinate.*—Of the different methods used to remove a portion of the middle turbinate, my preference is decidedly for the cold wire snare. The snare which is most desirable to me is the old Sajous slightly modified. I show it as modified by Powers and Anderson of Richmond, Va., for me. The change made is a very much larger thumbscrew, thereby facilitating turning, and, second, two holes are made in the end to contain the ends of the wire. This very greatly facilitates threading by putting one end of the wire through the hole farthest from the end and screwing it down until this passes within the shaft, when the remaining end of the wire may be passed into the distal opening, after which the excess may be cut with the wire scissors.

The next question is that of the most desirable local anesthetic. Certainly cocain secures for us the most profound anesthesia, but it greatly exsanguinates the soft tissue covering the bone, and therefore makes grasping it with the snare far more difficult. The shrinking of the lower turbinate by the action of cocain is, of course, desirable, since it produces a much larger cavity in the region of the lower turbinate and thereby facilitates the manipulation of the snare. Recently I have discontinued the use of cocain and have been using extract, a suprarenal capsule and holocain. After having made an application of an aqueous solution of suprarenal extract to the lower turbinate and inferior meatus, we secure as much shrinkage of the mucous membrane as it is possible to obtain from any known agent. After having applied the suprarenal extract we can effect satisfactory anesthesia of the middle turbinate by the use of holocain, which does not exsanguinate this body as cocain does. While holocain is quicker in its action than cocain, it is less profound in its effect, though my experience with it has been confined to weaker solutions than that of cocain which we ordinarily employ. Not being as familiar with the constitutional manifestations of holocain as with those of cocain has prompted me to use the weaker solution, and two per cent. has been the strongest application I have employed so far. By using the methods of anesthetizing just described it is possible to remove portions of the offending turbinal which it is impossible to do with the snare under cocain anesthesia. When the turbinate is enlarged throughout its extent it is not possible to surround it at one grasp of the snare. Under such circumstances it is most practicable to remove the posterior extremity first, and a second attempt may be made for the anterior portion. It is usually advisable, however, to leave the second portion for a subsequent sitting. Snaring the middle turbinate cannot be regarded as an easy operation, and occasionally we find it impossible to accomplish it. In such cases the biting forceps devised by Lester are invaluable. The wound is not left as smooth as it is from the cold snare, and, furthermore, it is sometimes necessary to make several "bites" before we remove it sufficiently.

Usually the hemorrhage ceases in a few minutes, except a slight tinge to the nasal secretions. When the hemorrhage is slightly excessive it is checked by a small pledget of cotton packed against the wound. Very rarely it is necessary to plug the nose tightly with iodoform or some antiseptic gauze, which must be removed on the following day, when it may or may not require a second packing. Secondary hemorrhage occasionally occurs, which demands the same management as does the primary bleeding.

The subsequent treatment consists in cleanliness, secured by some antiseptic spraying solution to be used by the patient. Infection has occasionally occurred, but its origin has practically always been traced to the lack of proper care. I have not seen any unfortunate complication result from infection other than a muco-purulent discharge accompanied by a certain degree of reaction in the region of the wound. My method of treating this complication is irrigation with a warm antiseptic solution, through the opposite nostril. I never remove both bones at the same sitting, but wait for the reaction produced by the first to subside before attacking the second.

